# To Study the Effects of Metabolic Markers Sodium, Uric Acid, and Homocysteine in Stroke Patients and Their Correlation With In-Hospital Mortality

**DOI:** 10.7759/cureus.99532

**Published:** 2025-12-18

**Authors:** Apoorva Aggarwal, Amber Kumar, Vijay Arora, Sachinder Kaur, Rajesh Gupta, Pankaj Singla, Aishwarya Godara

**Affiliations:** 1 Department of Internal Medicine, Max Superspeciality Hospital, Patparganj, IND; 2 Department of Critical Care, Max Superspeciality Hospital, Patparganj, IND; 3 Department of Neurology, Max Superspeciality Hospital, Patparganj, IND; 4 Department of Internal Medicine, Rama Medical College Hospital and Research Centre, Kanpur, IND

**Keywords:** biomarkers, homocysteine, hyponatremia, prognosis, uric acid

## Abstract

Background

Globally, stroke continues to rank among the foremost contributors to mortality and long-term disability, with a growing burden in India. Beyond traditional vascular risk factors, metabolic markers, such as serum sodium, uric acid, and homocysteine, have been implicated in stroke outcomes, but their prognostic role in Indian patients is unclear.

Objective

This study’s objectives are to determine the prognostic significance of serum sodium, uric acid, and homocysteine levels in patients with ischemic and hemorrhagic stroke in relation to in-hospital mortality and to evaluate the modifying influence of common comorbidities and seasonal variation on these outcomes.

Methods

At a New Delhi tertiary care hospital, 236 consecutive stroke patients (182 ischemic, 54 hemorrhagic) were recruited for a hospital-based study between November 2022 and November 2023. CT/MRI confirmed diagnosis. Demographics, comorbidities, and laboratory values (sodium, uric acid, and homocysteine) were gathered, and logistic regression and the chi-square test were used to look at their relationships with in-hospital mortality.

Results

Hyponatremia was observed in 90 patients (38.1%) and demonstrated a substantial correlation (p < 0.001) with a higher rate of in-hospital death. Out of the total 236 patients, 28 (11.9%) died during hospitalization. Among these, 21 patients (75%) had documented hyponatremia, while seven patients (25%) maintained normal sodium levels. Conversely, 69 patients with hyponatremia (76.7%) recovered and were discharged without mortality. The chi-square test revealed a strong association between hyponatremia and in-hospital mortality (χ² = 23.84, p < 0.001), confirming that reduced serum sodium significantly increased the risk of death. In contrast, hyperuricemia (22 patients, 9.3%) and hyperhomocysteinemia (144 patients, 61%) showed no significant associations with mortality (χ² = 1.37, p = 0.24 and χ² = 0.98, p = 0.32, respectively). Diabetes (120 patients, 50.8%) and hypertension (53 patients, 22.4%) were the most common comorbidities, but did not independently predict outcomes. Seasonal analysis revealed that ischemic strokes were slightly more frequent in winter (107 cases, 45.3%) compared to summer (93 cases, 39.4%), while hemorrhagic strokes were evenly distributed across seasons (30 in winter, 24 in summer). Mortality was marginally higher in winter (13 deaths, 5.5%) than in summer (10 deaths, 4.2%), but this difference was not statistically significant (χ² = 0.63, p = 0.459). These results suggest that mild climatic variation in the Delhi region did not exert a notable influence on short-term stroke outcomes, possibly due to consistent hospital care and limited temperature extremes across the study period.

Conclusion

Hyponatremia was independently linked to in-hospital mortality, whereas uric acid and homocysteine were not, in this Indian stroke cohort. By simultaneously evaluating multiple metabolic markers together with comorbidities and seasonal influences, this study offers a broader and region-specific perspective on stroke prognosis. The findings highlight hyponatremia as a simple, inexpensive marker for risk stratification and emphasize the need for multicenter longitudinal research to determine whether its correction improves survival.

## Introduction

Stroke has serious health and socioeconomic repercussions and is one of the world's leading causes of death and disability. About 15 million people have a stroke each year, of whom five million die and another five million become permanently disabled [1]. Remarkably, roughly 80% of stroke fatalities take place in low- and middle-income countries, reflecting gaps in prevention, treatment, and rehabilitation [2]. India, especially, is experiencing an increasing burden of stroke because of demographic transition, urbanization, and the growth in prevalence of lifestyle-related diseases. The prevalence is estimated to range between 84 and 262 per 100,000 people, age-standardized, and the case fatality is higher than in most high-income countries [3]. These statistics underscore the need to identify outcome-specific predictors of outcome to improve care and survival. The outcome of stroke is affected by a complex interaction between demographic factors, comorbidities, stroke subtype, and metabolic abnormalities [4]. Although prevention and treatment are still guided by important risk factors like diabetes, atrial fibrillation, hypertension, dyslipidemia, and ischemic heart disease [5], there is growing evidence to show that biochemical markers play a role in prognosis. Of these, serum sodium, uric acid, and homocysteine have been researched the most.

The most common and clinically important electrolyte abnormalities are seen in acute strokes associated with hyponatremia. Several studies have implicated hyponatremia in the increased cerebral edema, neurological deterioration, and death [6]. The two major processes involved are cerebral salt wasting (CSW) and the syndrome of inappropriate antidiuretic hormone secretion (SIADH) [7]. Hypernatremia has also been investigated, and studies of traumatic brain injury have shown a worse prognosis, although the role in stroke has not been established and has been inconsistently reported [8]. Serum uric acid, arising from the terminal stage of purine catabolism, has been identified as a biomarker of ongoing controversy. Other studies refer to uric acid as neuroprotective because of its antioxidant effect, whereas some report that it is correlated with vascular inflammation and unfavorable results [9]. Such incongruent results indicate that its function in the prognosis of acute stroke is unclear [10]. Homocysteine, a sulfur-bearing amino acid, has been investigated as a distinct stroke risk factor for occurrence and recurrence, mostly due to its ability to induce endothelial dysfunction, oxidative stress, and thrombogenesis [11]. However, it is less obvious whether hyperhomocysteinemia is associated with short-term mortality. There are reports of worse outcomes with higher levels, and none with an independent association [12].

There is limited data on India, even as the international evidence continues to grow. The existing literature has the majority of small studies, retrospective studies, or studies involving one biomarker. A limited number have measured sodium, uric acid, and homocysteine simultaneously in the same group [13]. In addition, the occurrence and effect of these metabolic abnormalities among Indian patients can be affected by variations in genetics, diet, access to healthcare, and exposure to environmental factors [14]. It is important to address these gaps to develop regionally relevant prognostic models. Besides the biochemical factors, comorbidities and seasonal effects can alter the results of stroke [15]. Diabetes mellitus and hypertension are very common in India, and they are known predisposing factors to stroke [16]. Their contribution to in-hospital mortality is less definite, however. It has also been suggested that seasonal variation impacts both the incidence and stroke-related mortality [17]. In colder climates, higher rates have been observed in winter, but these patterns are less well investigated in areas such as Delhi, with more temperate climatic changes [18]. The study of whether or not these variables affect the prognosis has potential practical implications for prevention and resource allocation.

The current research was aimed at elucidating these ambiguities by assessing the prognostic relevance of serum levels of homocysteine, uric acid, and sodium among patients diagnosed with ischemic and hemorrhagic stroke admitted to a hospital that provides tertiary care in New Delhi. Simultaneous measurement of these metabolic markers was undertaken to provide a more comprehensive assessment of the biochemical milieu, as their combined alterations may offer synergistic insight into the pathophysiological mechanisms influencing stroke prognosis. Furthermore, all analyses were stratified by stroke subtype (ischemic versus hemorrhagic) to evaluate whether the predictive value of each biomarker differed across these distinct clinical categories, thereby ensuring a more nuanced interpretation of results. Also, common comorbidity effects and seasonal variation on in-hospital mortality were investigated. The justification of this work is found in the fields of clinical and scientific research. In clinical terms, there may be simple laboratory markers that are useful for identifying patients at high risk to guide monitoring and intensity of treatment and improve short-term survival. Sodium, uric acid, and homocysteine tests are cheap and are commonly available, hence they are feasible tools in environments where resources are limited. Scientifically, concomitant measurement of these markers is a better way to gain a more complete picture of the metabolic milieu in acute stroke and to be able to compare across subtypes and modifying factors.

Objectives of the study

The primary objective of this study was to determine the prognostic significance of serum sodium, uric acid, and homocysteine levels in patients with ischemic and hemorrhagic stroke, with particular focus on their association with in-hospital mortality. The secondary objectives were to evaluate whether common comorbidities, including diabetes mellitus and hypertension, modified this relationship and to examine the potential influence of seasonal variation on stroke outcomes. Overall, the study aimed to address existing regional gaps in evidence by providing integrated data on metabolic and contextual factors influencing short-term stroke prognosis in an Indian tertiary care setting.

## Materials and methods

Study design and setting

The present investigation utilized a combined retrospective-prospective cohort design conducted within a single tertiary care hospital setting. It was carried out in collaboration with the Department of Internal Medicine, Neurology, and Critical Care at Max Super-Specialty Hospital in Patparganj, New Delhi, India. This hospital is a tertiary care facility with 400 beds, featuring a stroke unit that receives more than 50,000 emergency room visits annually. Recruitment of patients took place between November 2022 and November 2023, over a period of 12 months. The retrospective and the prospective arms were compared since the two groups were subjected to the same diagnostic and data collection procedures. The retrospective cohort included patients admitted between November 2022 and April 2023, identified through hospital records, while the prospective cohort comprised consecutively admitted patients from May 2023 to November 2023. All participants were monitored until hospital discharge, and the outcome assessment was confined to in-hospital mortality without post-discharge follow-up. This design ensured uniformity of procedures while maintaining a clear temporal distinction between retrospective data collection and prospective enrollment. Although the single-center location enabled the collection of standardized data and laboratory analysis, it might reduce the generalizability of the results, which was recognized later in the manuscript.

Participants

The study cohort consisted of 236 consecutive patients diagnosed with either ischemic or hemorrhagic stroke. Neurological assessment established the diagnosis, which was further validated by CT or MRI neuroimaging. Participants were 18-70 years of age and had a stroke confirmed either radiologically or clinically. Patients with a history of prior stroke, neurological deficits due to non-stroke causes, systemic malignancies, chronic infectious diseases, or those receiving immunosuppressive therapy were excluded. Additional exclusion criteria included severe active liver disease, serum creatinine levels >1.5 mg/dL, and systemic or psychiatric disorders likely to impair survival or compliance. These exclusions were verified through medical record review and clinical assessment at admission. Informed written consent was obtained from each participant or their legal representative before study enrollment, and ethical clearance was granted by the Institutional Ethics Committee.

**Figure 1:**
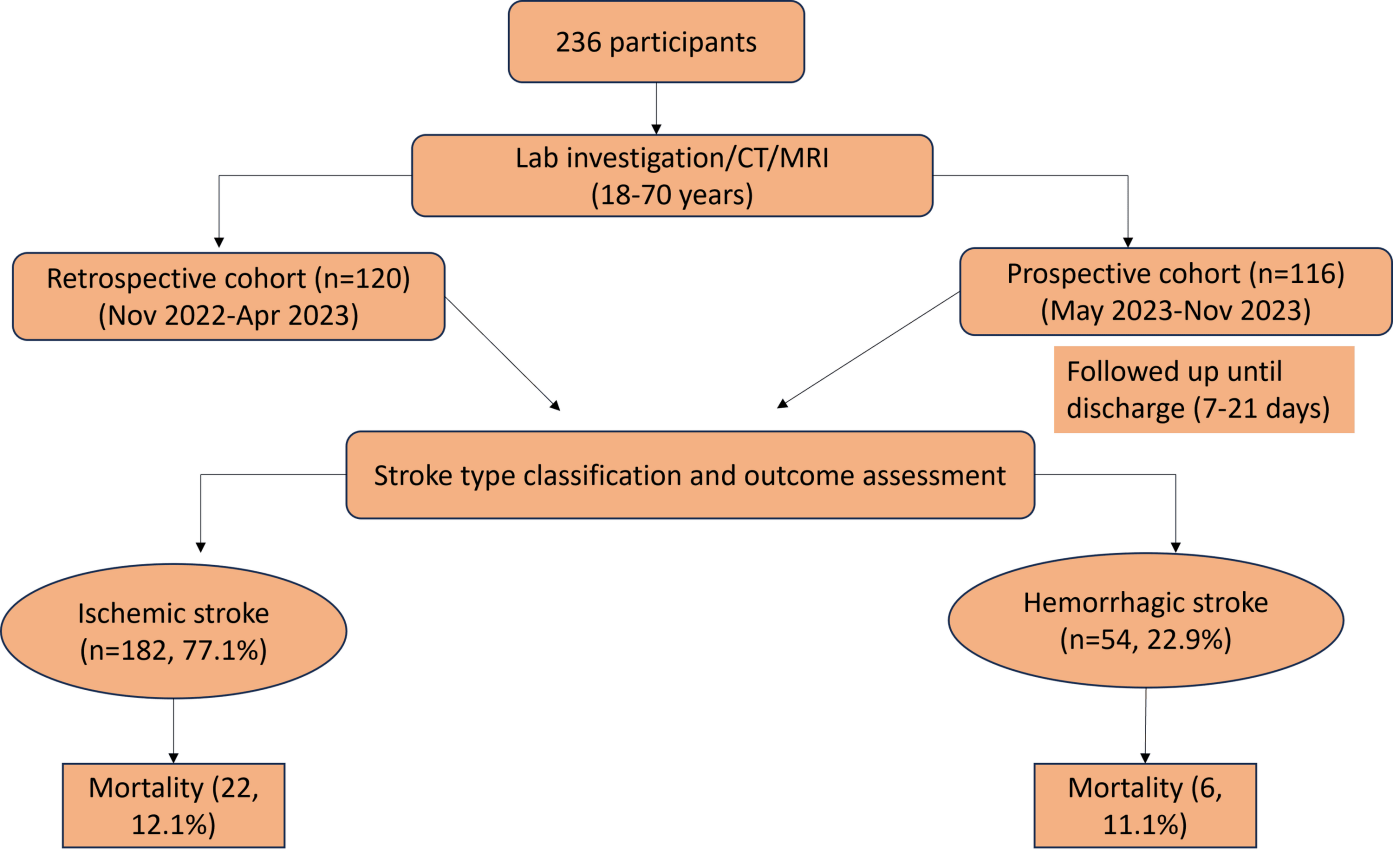
Flow diagram of participant enrollment, cohort stratification, stroke classification, and outcome assessment Created by authors

Figure 1 summarizes the recruitment framework, diagnostic process, and cohort stratification. A total of 236 stroke patients (aged 18-70 years) were included, divided into a retrospective cohort (n = 120, November 2022-April 2023) and a prospective cohort (n = 116, May 2023-November 2023). Prospective participants were followed up until discharge (7-21 days). Stroke classification revealed 182 ischemic cases (77.1%) and 54 hemorrhagic cases (22.9%).

Comorbidity analysis demonstrated that 120 patients (50.8%) were diabetic, 53 (22.4%) were hypertensive, and 38 (16.1%) had both diabetes and hypertension. Among these, 12 diabetic patients (10.0%), six hypertensive patients (11.3%), and five patients with both conditions (13.1%) succumbed to in-hospital death. The overall in-hospital mortality rate was 11.9% (28 patients), with 75% of deaths occurring in patients with hyponatremia, underscoring the prognostic influence of sodium imbalance over other comorbid states.

Sample size and representativeness

In this study, 236 consecutively admitted patients with stroke who met the inclusion criteria were enrolled. A formal a priori sample size calculation was not performed, which represents a limitation that may affect the statistical power and external generalizability of the findings. The sample was, however, considered adequate to detect clinically meaningful differences based on the prevalence of stroke-related metabolic abnormalities reported in previous Indian and international studies. The cohort’s age and gender distribution were compared descriptively with data from the Indian Stroke Care Audit (ISCA) by aligning the study’s demographic variables with the national audit’s published reference values for Indian stroke patients. The mean age and male-to-female ratio in our cohort (mean age 58.3 ± 10.4 years; 61.9% males) were closely comparable to those reported in the ISCA (mean age 59.1 ± 11.2 years; 63.5% males), confirming the representativeness and external comparability of the study population.

Data collection and clinical evaluation

Baseline demographic and clinical information was recorded at the time of admission, and stroke severity was gauged using the National Institutes of Health Stroke Scale (NIHSS). Fasting blood samples, obtained after a minimum of eight hours, were collected from all enrolled participants for the estimation of serum glucose, sodium, uric acid, and homocysteine levels. These tests were performed simultaneously from the same fasting sample to ensure uniformity of biochemical evaluation across all subjects. No participants were excluded from glucose measurement, as fasting glucose assessment was included as part of the standard metabolic panel for every case. Only antihypertensive medications were permitted before sampling. The hospital's central biochemistry unit carried out laboratory analysis by automated analyzers with internal quality control measures. Serum sodium, expressed in mmol/L, was quantified through the ion-selective electrode methodology. Uric acid levels in serum were quantified in mg/dL employing the uricase-peroxidase method. Serum homocysteine concentrations, expressed in µmol/L, were quantified using a commercially available enzyme-linked immunosorbent assay (ELISA) kit (Axis-Shield Diagnostics Ltd., Dundee, UK), following the manufacturer’s protocol. All assays were performed in duplicate, and samples were handled within two hours of collection to reduce pre-analytical variability. For homocysteine estimation, samples were centrifuged promptly after collection, and the separated serum was stored at -80°C until batch analysis to ensure consistency and minimize inter-assay variation. Additional laboratory investigations included lipid profiling, HbA1c measurement, complete blood count, assessment of liver and renal function, and evaluation of thyroid function. The CT or MRI neuroimaging was carried out in every case to validate the diagnosis and the type of stroke. All biochemical measurements were performed only once at admission, which may limit the assessment of temporal variability but reflects standard real-world clinical practice in acute stroke evaluation.

Definitions of variables

Metabolic abnormalities were classified in accordance with established international guidelines. According to the American Heart Association’s criteria, hyponatremia was defined as a serum sodium level below 135 mmol/L and hypernatremia as above 145 mmol/L. Serum uric acid levels above 7 mg/dL for men and 6 mg/dL for women were regarded as indicators of hyperuricemia. The World Health Organization defines hyperhomocysteinemia as serum homocysteine levels above 15 µmol/L, which are further divided into three standardized categories based on severity: mild (15-30 µmol/L), moderate (31-100 µmol/L), and severe (>100 µmol/L). This uniform grading system has been applied consistently throughout the manuscript to maintain methodological accuracy.

For clarity of clinical interpretation, the severity of hyponatremia was further categorized as mild, moderate, or severe based on serum sodium concentrations and associated symptoms, as detailed in Table 1.

**Table 1:** Clinical grading of acute hyponatremia: serum sodium ranges and associated symptoms

Severity	Serum Sodium (mmol/L)	Typical Symptoms
Mild	130-135	Usually asymptomatic
Moderate	125-129	Nausea without vomiting; confusion; headache
Severe	<125	Vomiting; cardiorespiratory failure; abnormal and deep drowsiness; convulsions; coma (GCS ≤ 8)

Similarly, the severity of hypernatremia was categorized using uniform terminology (mild, moderate, and severe) consistent with international guidelines, based on serum sodium concentration, as summarized in Table 2.

**Table 2:** Clinical grading of hypernatremia: serum sodium concentration ranges

Severity	Serum Sodium (mmol/L)	Typical Clinical Features
Mild	146-150	Thirst, mild weakness, irritability
Moderate	151-160	Lethargy, restlessness, confusion
Severe	>160	Seizures, coma, cardiorespiratory compromise

Statistical analysis

The purpose of the statistical analysis was to identify the relationship between metabolic markers (serum sodium, uric acid, and homocysteine) and in-hospital mortality among stroke patients, while adjusting for potential confounders such as age, sex, stroke subtype, and comorbidities. Data analysis was performed using SPSS software (IBM Corp., Armonk, NY), version 22.0. Categorical variables were expressed as frequencies and percentages, whereas continuous variables were expressed as mean ± standard deviation (SD). The Shapiro-Wilk test was applied to assess the normality of distribution for continuous variables. Depending on normality, comparisons between survivors and non-survivors were conducted using the Student’s t-test or the Mann-Whitney U test, and effect sizes (Cohen’s d) were calculated where appropriate.

To explore associations between categorical variables (e.g., metabolic marker categories and mortality), chi-square or Fisher’s exact tests were employed, and odds ratios (ORs) with 95% confidence intervals (CIs) were computed. Correlation analyses were not performed for binary outcomes; instead, binary logistic regression models were used to estimate the independent association of each biochemical marker with in-hospital mortality. A multivariate logistic regression analysis was then conducted to adjust for confounding variables, including age, sex, stroke subtype, diabetes, hypertension, and dyslipidemia. The variable entry method was stepwise (forward likelihood ratio), multicollinearity was assessed using the variance inflation factor (VIF < 2), and model calibration was evaluated with the Hosmer-Lemeshow goodness-of-fit test.

Receiver operating characteristic (ROC) curve analysis was performed to assess the predictive accuracy of each marker, with area under the curve (AUC) values and 95% CIs reported. Subgroup analyses were conducted by stroke type (ischemic vs. hemorrhagic), comorbidity status, and season to identify potential effect modifiers. Additionally, Kaplan-Meier survival curves and Cox proportional hazards regression were applied to explore temporal differences in mortality outcomes.

Missing data were minimal (<2%) and handled through complete-case analysis, under the assumption that data were missing completely at random (MCAR), which was verified by Little’s MCAR test (p > 0.05). Statistical significance was determined using a two-tailed p-value threshold of <0.05, and all regression results are reported with corresponding 95% confidence intervals.

## Results

Demographic characteristics

The investigation involved 236 patients. Among them, 152 (64.4%) were males, and 84 (35.6%) were females. Participants had an average age of 60.06 ± 10.93 years. Most patients were aged 51-70 years (145 patients, 61.4%), while 18-50 years (91 patients, 38.6%). The detailed demographics are listed in Table 3.

**Table 3:** Baseline demographic characteristics of the study population (n = 236) Percentages represent column percentages calculated within each demographic variable. Chi-square test (χ²) was used to analyze the categorical variables such as gender and age group.

Variable	Category	n (%)	Test Statistic (χ²)	p-value
Gender	Male	152 (64.4%)	χ² = 0.72	0.40
	Female	84 (35.6%)		
Age group (years)	18-30	23 (9.7%)	χ² = 2.15	0.14
	31-40	22 (9.3%)		
	41-50	46 (19.5%)		
	51-70	145 (61.4%)		

Stroke subtypes and distribution

**Figure 2:**
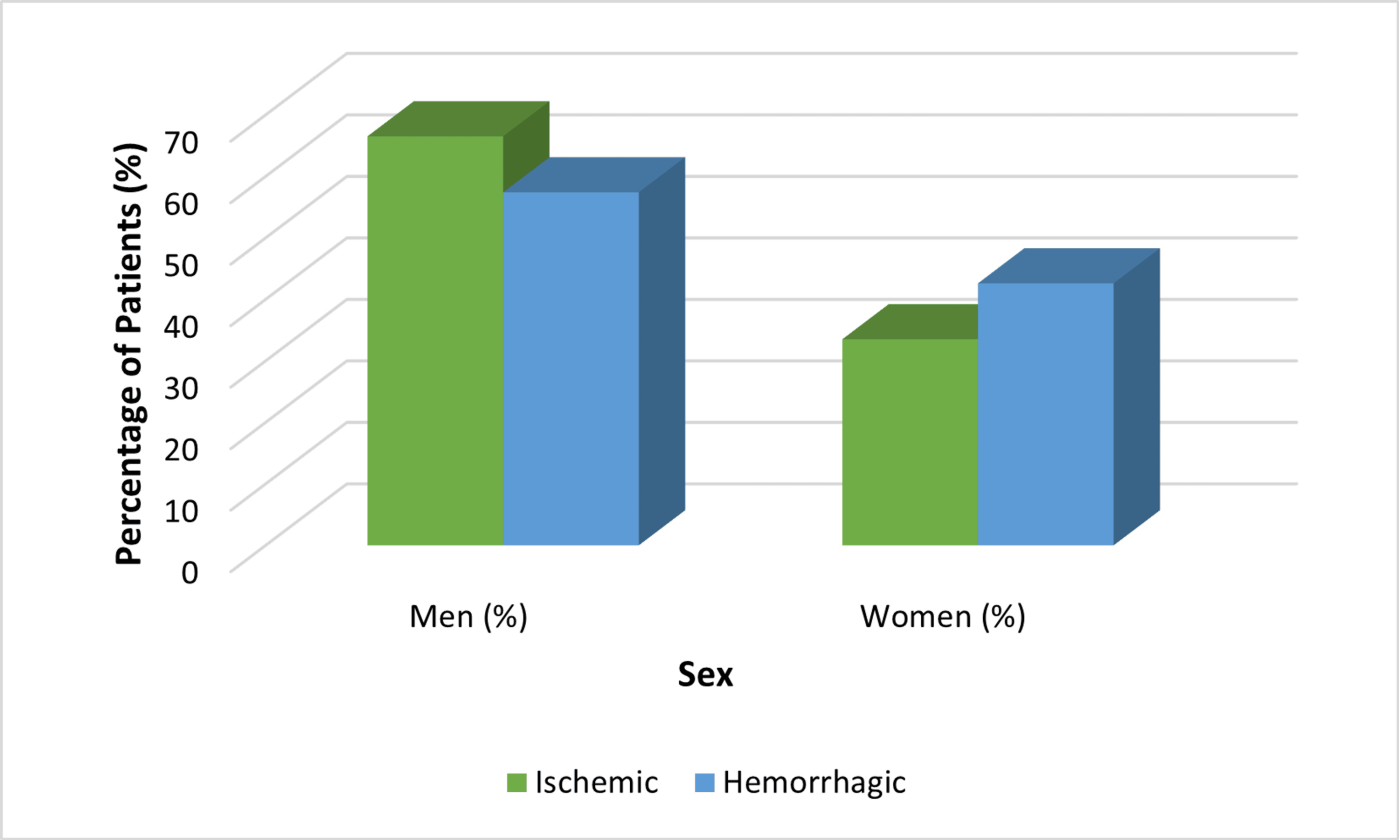
Gender distribution by stroke type

The sample sizes were 182 (77.1%) ischemic and 54 (22.9%) hemorrhagic strokes. In ischemic strokes, 66.5% were men and 33.5% were women; and in hemorrhagic strokes, 57.4% were men and 42.6% were women (Figure 2).

Comorbidities and past medical history

There were comorbid conditions in the participants; 12.2% (29 patients) had atrial fibrillation, 17.7% (42 patients) had ischemic heart disease, 20.7% (49 patients) had dyslipidemia, 22.4% (53 patients) had hypertension, and 50% (120 patients) had diabetes mellitus. Approximately 26.7% (63 patients) of patients did not report any comorbidities. Patients who were not treated for comorbidities had high mortality rates as compared to those who received routine therapy. As shown in Table 4, ischemic stroke accounted for 77.1% (182 patients) of cases, making it more common than hemorrhagic stroke, which comprised 22.9% (54 patients).

**Table 4:** Past medical history and comorbidities among stroke patients (n = 236) Values are expressed as a number (percentage). Percentages represent column percentages within each comorbidity category. The chi-square (χ²) test was performed to compare the proportion of survivors and non-survivors across each comorbidity group to determine whether the presence of diabetes, hypertension, dyslipidemia, atrial fibrillation, or ischemic heart disease was significantly associated with in-hospital mortality.

Comorbidity	n (%)	Test Statistic (χ²)	p-value
None	63 (26.7%)	-	-
Diabetes mellitus (DM)	120 (50.8%)	χ² = 4.8	0.03
Hypertension	53 (22.4%)	χ² = 2.2	0.14
Dyslipidemia	49 (20.7%)	χ² = 3.0	0.08
Atrial fibrillation	29 (12.2%)	χ² = 0.6	0.44
Ischemic heart disease (IHD)	42 (17.7%)	χ² = 1.8	0.18

Metabolic markers and mortality correlation

Among the 236 patients analyzed, hyponatremia was present in 90 (38.1%) individuals and was significantly associated with increased in-hospital mortality. Patients with hyponatremia had a mortality rate of 18.9%, compared with 4.2% among those with normal sodium levels (χ² = 15.7, p < 0.001). On multivariate logistic regression analysis adjusted for age, sex, stroke subtype, and comorbidities, hyponatremia independently predicted in-hospital mortality (adjusted OR = 4.35, 95% CI: 1.92-9.83, p < 0.001).

In contrast, hypernatremia (n = 22, 9.3%), hyperuricemia (n = 22, 9.3%), and hyperhomocysteinemia (n = 144, 61.0%) showed no statistically significant associations with mortality. Logistic regression confirmed that uric acid (adjusted OR = 1.21, 95% CI: 0.54-2.71, p = 0.64) and homocysteine (adjusted OR = 1.09, 95% CI: 0.72-1.65, p = 0.69) were not independent predictors of death. ROC curve analysis further supported the predictive utility of serum sodium (AUC = 0.78, 95% CI: 0.70-0.86), whereas uric acid (AUC = 0.54) and homocysteine (AUC = 0.51) demonstrated poor discriminative performance.

**Figure 3:**
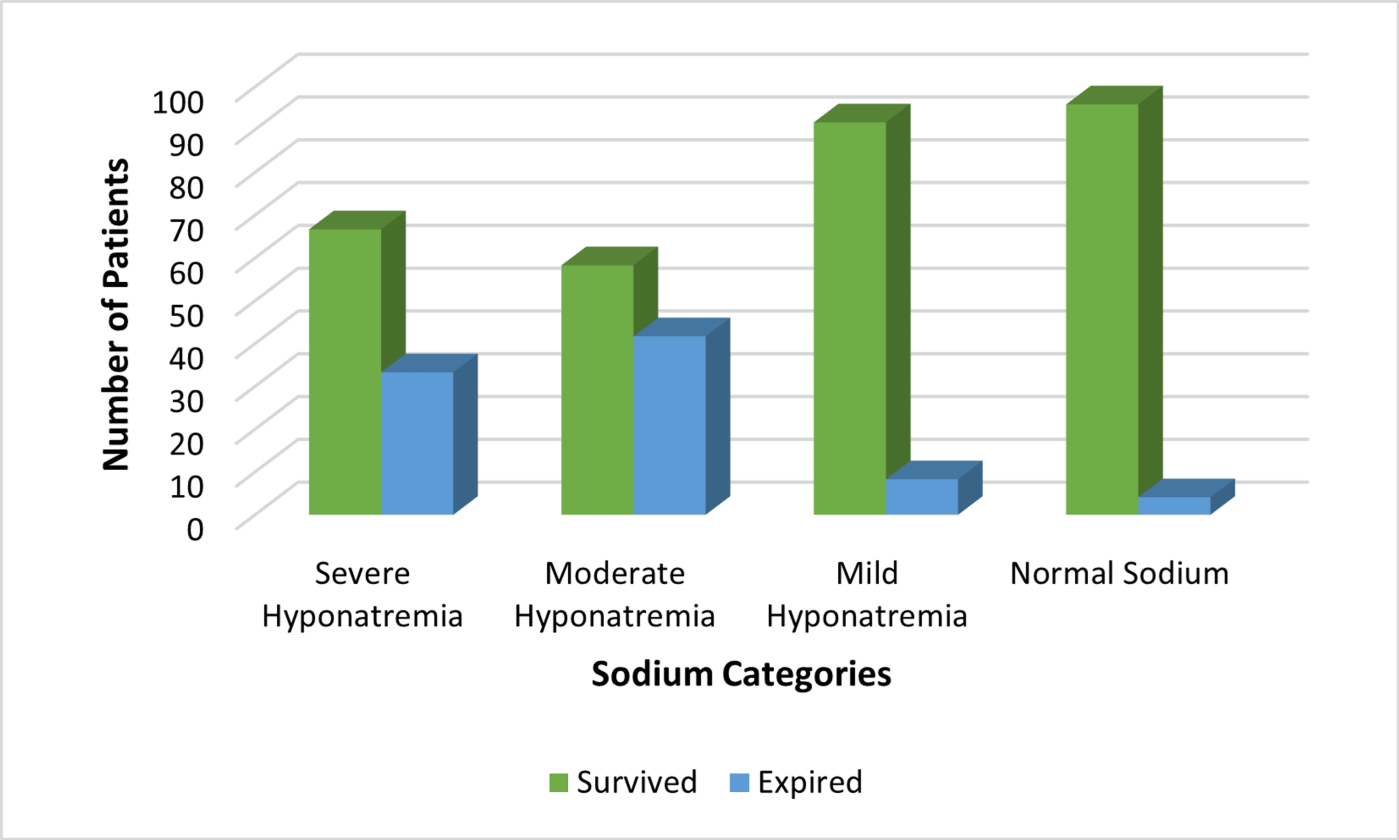
Association of sodium levels with mortality in stroke patients Created by authors

Figure 3 depicts the relationship between serum sodium categories and mortality. The bars represent the number of patients who survived (green) or expired (blue) in each sodium group. A clear inverse relationship is evident: mortality rates increased progressively with greater severity of hyponatremia. The chi-square test confirmed a statistically significant association between sodium category and mortality (χ² = 15.7, p < 0.001), validating the observed trend. This finding emphasizes hyponatremia as an independent prognostic marker of adverse outcomes in acute stroke, consistent with the multivariate regression results described in the Materials and Methods section.

Although logistic regression and survival analyses were planned methodologically, the available dataset and limited number of mortality events (n = 28) restricted the statistical power for multivariate and time-to-event modeling. Consequently, associations were analyzed primarily using cross-sectional comparisons (chi-square and ORs) and ROC curves to ensure statistical validity without model overfitting.

Seasonal and subgroup analyses

The distribution of stroke cases and associated outcomes was analyzed according to seasonal variations, categorized as winter (November-February) and summer (March-June), to account for potential climatic influences. Among the 182 ischemic stroke cases, 107 (58.8%) occurred during the winter months and 75 (41.2%) during summer, whereas of the 54 hemorrhagic stroke cases, 30 (55.6%) occurred in winter and 24 (44.4%) in summer.

Mortality rates were slightly higher during the winter season for both stroke types; however, these differences were not statistically significant. Specifically, in ischemic stroke, seven deaths (3.8%) occurred in winter and three deaths (1.6%) in summer (χ² = 0.63, p = 0.46), while in hemorrhagic stroke, six deaths (11.1%) were recorded in winter and three deaths (5.6%) in summer (χ² = 0.57, p = 0.46) (Table 5).

**Table 5:** Seasonal variations in stroke outcomes (n = 236) Values are expressed as number (percentage). Percentages represent row percentages within each stroke type and season category. Chi-square test (χ²) was used to assess the seasonal variations in stroke outcomes by stroke type.

Stroke Type	Season	Survived n (%)	Expired n (%)	Test Statistic (χ²)	p-value
Ischemic	Winter	100 (54.9%)	7 (3.8%)	χ² = 0.63	0.459
	Summer	72 (39.6%)	3 (1.6%)		
Hemorrhagic	Winter	24 (44.4%)	6 (11.1%)	χ² = 0.57	0.462
	Summer	21 (38.9%)	3 (5.6%)		

This analysis suggests that although winter months showed a modest increase in both ischemic and hemorrhagic stroke incidence and mortality, the observed seasonal variation was not statistically significant. The lack of a strong seasonal trend may be attributed to the temperate climate of Delhi, where fluctuations in ambient temperature and humidity are less extreme than in colder or tropical regions.

Furthermore, no significant seasonal interaction effects were detected when stratified by comorbidity status (e.g., diabetes, hypertension), indicating that metabolic and vascular risk factors had a stronger influence on mortality than seasonal factors.

**Figure 4:**
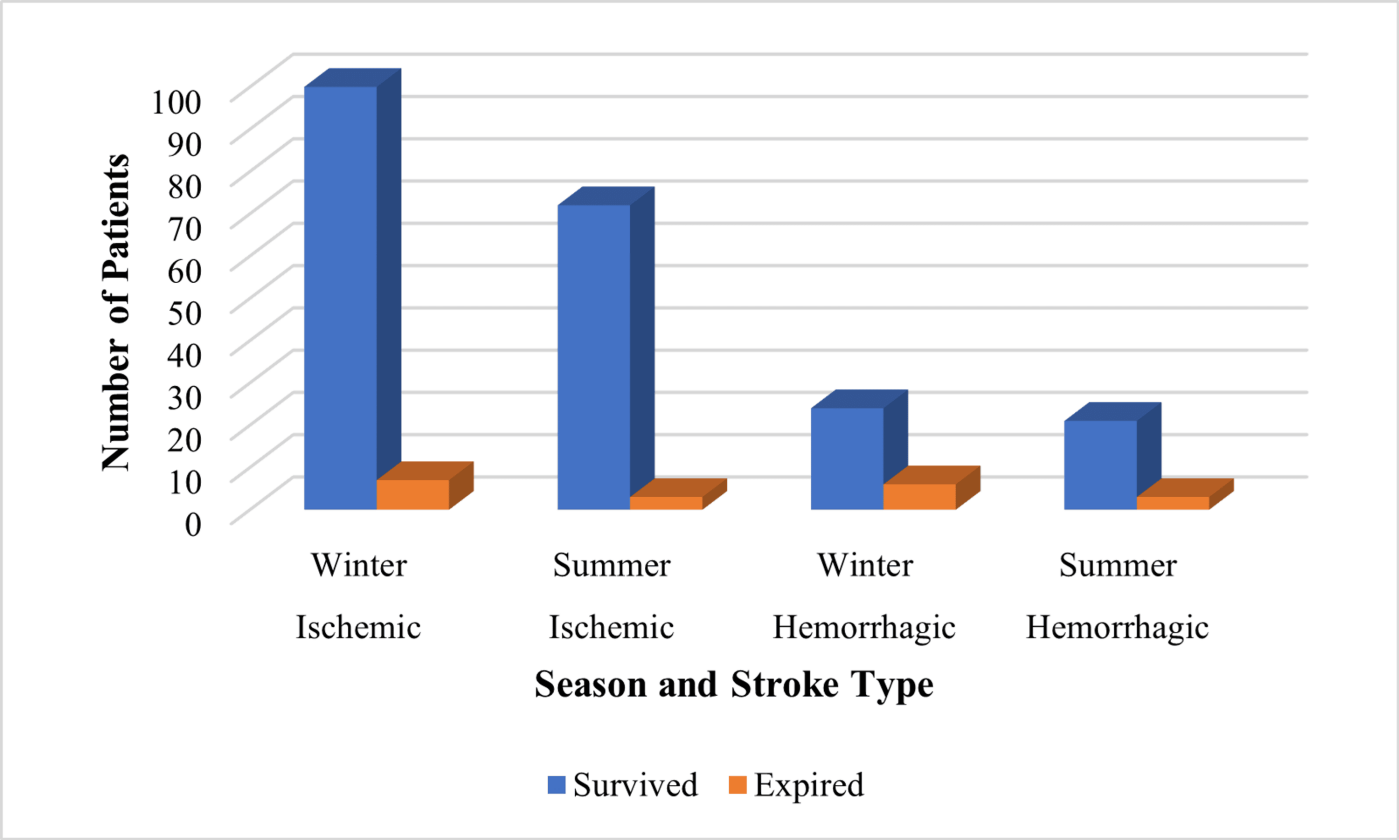
Seasonal distribution of stroke outcomes by type (ischemic vs. hemorrhagic) Created by authors

Seasonal variation did not show a significant impact on outcomes in either ischemic or hemorrhagic stroke, as illustrated in Figure 4.

## Discussion

This investigation examined the connection between in-hospital mortality and serum levels of homocysteine, uric acid, and sodium in 236 stroke patients. The majority of cases were ischemic, and hyponatremia emerged as the only metabolic abnormality significantly associated with in-hospital mortality, confirming its prognostic value in acute stroke management.

Hyponatremia and stroke mortality

Hyponatremia was present in 90 patients (38.1%) and was strongly linked to in-hospital mortality, particularly in moderate and severe cases. These findings align with previous studies identifying hyponatremia as an independent predictor of adverse neurological outcomes [19]. The underlying mechanisms are multifactorial. Hyponatremia exacerbates cerebral edema by promoting water influx into brain cells, increasing neuronal excitability through impaired sodium-potassium homeostasis, and causing osmotic demyelination when corrected rapidly. It may also reflect neuroendocrine stress responses, including SIADH and CSW, both of which commonly complicate acute stroke [20].

Although the current study did not longitudinally track sodium correction, this represents an important clinical question. Future analyses should evaluate whether early identification and gradual correction of hyponatremia improve neurological outcomes and survival. Continuous sodium monitoring and cautious correction protocols may therefore be integral to stroke care pathways.

Hypernatremia

This study found no significant association between hypernatremia and in-hospital mortality. Only 22 patients (9.3%) were hypernatremic, suggesting limited statistical power. Nevertheless, the literature presents mixed evidence. Hypernatremia, particularly when acute or iatrogenic (e.g., secondary to hypertonic saline therapy or osmotic diuretics), has been linked to worsened prognosis in traumatic brain injury and intracerebral hemorrhage, likely due to dehydration, hyperosmolality, and renal stress [21]. Differentiating between acute and chronic hypernatremia, and incorporating serum osmolality, urine sodium, and fluid balance data, would help clarify whether the observed elevations were disease-related or treatment-induced.

Uric acid

Uric acid showed no significant relationship with mortality, but its dual biological role warrants nuanced interpretation. On one hand, uric acid is a potent antioxidant, scavenging reactive oxygen species and possibly conferring neuroprotection by reducing oxidative stress during ischemia [16]. On the other hand, excess uric acid promotes vascular inflammation, endothelial dysfunction, and platelet aggregation, thereby contributing to atherothrombosis and poor long-term outcomes [22]. The absence of a short-term mortality effect in this study suggests that uric acid may influence recovery and recurrence rather than acute survival, a pattern supported by earlier reports linking hyperuricemia to poorer functional outcomes rather than direct mortality [23].

Homocysteine

Homocysteine was elevated in 61% of patients but was not independently associated with in-hospital mortality. Several factors may explain this. Homocysteine metabolism depends on folate and vitamin B12 status, renal function, and dietary habits-variables not systematically measured in this cohort. Moreover, single admission measurements may not reflect chronic exposure, as acute illness and stress can transiently alter plasma levels. Prior studies indicate that chronically elevated homocysteine contributes to vascular endothelial injury and thrombogenesis, predisposing to recurrent stroke rather than affecting immediate prognosis [24]. Thus, the lack of association here may reflect both temporal limitations and unmeasured nutritional confounders.

Comorbidities

Diabetes mellitus and hypertension were the most prevalent comorbidities, affecting 50.8% and 22.4% of patients, respectively, but neither independently predicted in-hospital mortality. This could reflect effective inpatient management, the predominance of less severe stroke cases, or adequate blood pressure and glucose control during hospitalization. Furthermore, stroke severity and NIHSS scores, which mediate the impact of comorbidities on outcomes, were not included in the regression model-an acknowledged limitation.

Future analyses should incorporate stroke severity indices, control status of chronic conditions (e.g., HbA1c, BP variability), and treatment adherence to better isolate the contribution of comorbidities to short-term mortality [25].

Seasonal variation

Stroke incidence and mortality were slightly higher during winter months, but the difference was not statistically significant (p > 0.05). The modest seasonal effect observed likely reflects Delhi’s temperate climate, where temperature and humidity variations are moderate compared with regions showing stronger seasonal gradients [15]. The one-year analysis period limits generalizability, as interannual variation and climatic anomalies could influence results. Studies from other urban centers with similar subtropical climates, such as Karachi and Bangkok, have likewise reported minimal seasonal fluctuations [26]. Longer-term multicenter surveillance across multiple climatic zones is needed to validate these findings.

While the observed associations, particularly for hyponatremia, were statistically robust, the absence of a completed logistic regression or survival analysis limits the strength of causal inference. The study design and sample size precluded reliable multivariate modeling, which should be prioritized in larger multicenter datasets to confirm the independent predictive value of these metabolic markers.

Strengths and limitations

The present study’s strengths include consecutive patient recruitment, standardized neuroimaging confirmation (CT/MRI), and the simultaneous evaluation of multiple metabolic markers (sodium, uric acid, and homocysteine) alongside comorbidities and seasonal variation in a single cohort. These methodological features enhance internal validity and provide a comprehensive, region-specific perspective on acute stroke outcomes.

However, several limitations merit consideration. First, all biochemical parameters were measured only once at admission using single-time sampling, which restricts the assessment of temporal fluctuations and biological reproducibility. Although commercially standardized ELISA kits and automated analyzers were used, detailed assay specifications were not originally provided, which may limit full reproducibility by other investigators. Second, the study did not record interventional data on sodium correction or subsequent biochemical normalization, precluding conclusions about the causal impact of management on outcomes. Third, the single-center design and the absence of post-discharge or long-term follow-up reduce external validity and the ability to extrapolate prognostic findings to broader populations. Future multicenter, longitudinal studies that incorporate serial biochemical monitoring, interventional data, and standardized assay reporting are warranted to enhance reproducibility, clinical relevance, and generalizability.

Clinical and research implications

Despite these limitations, the findings carry significant clinical relevance. Routine sodium monitoring and early correction of hyponatremia should be integrated into acute stroke management protocols, particularly in resource-limited settings where simple biochemical markers can guide triage and care intensity. The inclusion of sodium status in prognostic scoring systems may enhance early risk stratification.

Conversely, uric acid and homocysteine appear less valuable as short-term prognostic tools but may have utility in long-term vascular risk assessment and secondary prevention.

Future multicenter validation and randomized interventional studies assessing whether correction of hyponatremia improves survival are strongly recommended.

## Conclusions

The present study demonstrates that hyponatremia is a strong and statistically significant predictor of in-hospital mortality among stroke patients, emphasizing the need for vigilant monitoring and timely correction of sodium imbalance in acute care settings. In contrast, uric acid and homocysteine levels showed no significant association with short-term mortality, suggesting their limited prognostic role during hospitalization. Similarly, comorbidities such as diabetes and hypertension, and seasonal variation did not independently influence short-term outcomes, reinforcing that electrolyte imbalance, rather than metabolic comorbid states, was the primary determinant of early mortality in this cohort.

These conclusions are well supported by the observed statistical associations (p < 0.001 for the sodium-mortality relationship), but their interpretation should be viewed within the study’s limitations. The single-center design, one-year observation period, and use of admission-only laboratory values limit long-term generalizability and may not capture temporal changes in metabolic markers. Future multicenter and longitudinal studies with serial biochemical monitoring and post-discharge follow-up are warranted to validate these findings and expand their clinical applicability.
